# Sadder but Wiser: The Role of SARS Imprinting and Firms’ Recovery During the COVID-19 Pandemic

**DOI:** 10.3389/fpsyg.2022.917337

**Published:** 2022-06-10

**Authors:** Haitang Yao, Jiayang Wang, Qingwen Bo, Mingliang Li

**Affiliations:** ^1^Business School, Qingdao University, Qingdao, China; ^2^School of Management, Harbin Institute of Technology, Harbin, China; ^3^School of International Business, Tianjin Foreign Studies University, Tianjin, China; ^4^Khorgas Business School, Yili Normal University, Yining, China

**Keywords:** COVID-19, SARS, recovery, organizational imprinting, digitalization

## Abstract

Our study provides a new research perspective on firms’ recovery during the COVID-19 pandemic, i.e., can similar events experienced by firms in the past have an imprinting effect on the improvement of firm’s recovery? We focus on firms’ Severe Acute Respiratory Syndromes (SARS) imprints. Based on four quarters of panel data of Chinese A-share listed companies in 2020, our study finds that SARS imprints are positively related to firms’ recovery ability during the COVID-19 pandemic. Meanwhile, if the more severe the SARS pandemic experienced by a firm, the more significant the effect of SARS imprint on the firm’s recovery ability during the COVID-19 pandemic. In addition, the higher the level of digitization of firms during the COVID-19 pandemic, the more it contributed to the enhancing effect of the SARS seal on firm recovery. Our study makes an important theoretical contribution to the recovery literature as well as to imprinting theory, while providing practical guidance for improving the recovery of firms during the COVID-19 pandemic.

## Introduction

The COVID-19 pandemic, one of the worst crises in human history, has caused global economic turmoil and a collapse in business activity. Due to its contagiousness and health risks, countries around the world have set up restrictions such as lockdown, close of ports and airports, strict manpower norms, and other restrictive measures. Lockdown policies have led to the downgrading of manufacturing, increased layoffs, increased unemployment, slowing demand, and declining industrial profits (Gössling et al., 2020; Nicola et al., 2020). Many industries such as aviation, hospitality, tourism, entertainment and transport are struggling to survive, and many enterprises are on the verge of closure. However, another group of enterprises were less affected by the COVID-19 pandemic because they were sufficiently crisis-resistant and recovered more quickly than other organizations even after being affected by the pandemic. It is evident that building recovery quickly is critical for firms, and the faster they act, the more likely they are to reduce the severity of losses and protect themselves from ongoing damage (Kong et al., 2021).

Through reading the research literature of scholars on firm recovery, we found that most scholars focus on how to rapidly increase turnover (DesJardine et al., 2019; Kettunen et al., 2021), increase supply chain sustainability (Sarkis, 2020), and respond faster (Jin et al., 2018; Kong et al., 2021), but all of this literature ignores the important fact that certain imprints of the organization can also have an impact on post-crisis recovery. Our study designs a framework in which we explain why a subset of firms recover faster from having previously experienced similar events (Dormady et al., 2022). In our framework, we argue that firms experienced the SARS event had faster recovery rates when the COVID-19 pandemic broke out compared to other firms that did not experience SARS.

Our study has several important contributions. First, we contribute to the recovery literature by exploring the relationship between SARS imprinting and firm recovery, particularly during the COVID-19 pandemic. There have been more studies that have explored the impact of pandemic COVID-19 on individual mental health and recovery from an individual perspective (Ahmed et al., 2020), however, there is still less research on firms’ recovery following the COVID-19 pandemic, with only a few studies looking at executive characteristics (Chesbrough, 2020), corporate strategy (Kong et al., 2021), technological capabilities (Doerr et al., 2021), but this literature ignores the impact of firm imprints. Our study empirically analyses the impact of SARS imprinting on firms’ recovery during the COVID-19 pandemic, filling part of the research gap.

Second, there is still a paucity of academic research on SARS imprinting, with only two papers in the existing literature that have examined SARS imprinting, one by Ru et al. (2021) for country-level SARS imprinting and the other by Yao et al. (2021) for individual-level SARS imprinting. To the best of our knowledge, no scholar has introduced SARS imprinting into firm-level studies prior to our study. In our framework, we argue that a firm is imprinted with the SARS imprint if it experienced the SARS pandemic in that year, providing new insights into the application of imprinting theory to the COVID-19 pandemic period and bridging the limitations of imprinting theory in the current crisis. That is, companies can find ways to recover from a COVID-19 pandemic by looking at past experiences of similar events.

## Theoretical Background and Hypothesis

### Imprinting Theory

Since Stinchcombe (1965) introduced “imprinting” to the field of organizations, the concept has been widely used at a number of levels, including organizational collectives, single organizations and organizational constructs, and has been extended to the study of supra-organizational entities such as industries and networks (Boari and Riboldazzi, 2014). Although most entities across levels of analysis can be the object of imprinting, researchers have primarily focused on organizational imprinting and, to a lesser extent, individuals. The initial insight into organizational imprinting was that the structure of a company reflected its founding environment, particularly the technology available at the time of founding (Stinchcombe, 1965). Much of the existing literature on organizational imprinting has focused on a variety of environmental imprints, including the imprinting effect of founding in a legitimacy vacuum (Dobrev and Gotsopoulos, 2010), the imprinting effect associated with the firm’s international expansion, the environment and conditions under which the firm was founded (Geroski et al., 2010), the firm system (Waeger and Weber, 2019), the imprinting effect of venture capital on firm performance (Croce et al., 2013), the legacy imprint of high-tech start-ups (Colombo and Piva, 2012), and the imprinting effect of the firm’s international expansion (Dobrev and Gotsopoulos, 2010; Colombo and Piva, 2012), etc. Specifically, Geroski et al. (2010) explored the impact of establishment conditions such as macroeconomic conditions, firm size, and industry concentration on the survival of new firms, and showed that the conditions and environment at the time of a firm’s birth are the largest influences on a firm’s chances of survival and appear to have a relatively long-term impact on survival. Waeger and Weber (2019) argue that a firm’s current institutional logic conforms, at least in part, to historical imprints developed in past environments, and that the importance of the imprints of firms’ historical political characteristics needs to be considered when examining contemporary manifestations of institutional complexity in firms. These studies all suggest that the environment is an important source of imprinting. That is, environmental conditions at the time of a firm’s founding have an ongoing impact on the firm’s survival, and the imprint of these particular circumstances can persist despite significant changes in the environment in subsequent periods (Marquis and Tilcsik, 2013).

Imprinting theory suggests that organizations or individuals go through a number of “sensitive periods” (Pieper et al., 2015) during their development, during which they focus on exhibiting a high degree of sensitivity to the external environment, allowing organizations or individuals to potentially develop imprinting characteristics with a particular environment. For individuals, what imprinting theory refers to as “sensitive periods” are not only the “early” experiences of individuals, but also periods that have shaped their worldview, values and outlook on life, or periods of individual role transitions, for example, the famine in China (Long et al., 2020), SARS experiences during childhood (Yao et al., 2021), Total Wealth Characteristics (Korkeamäki et al., 2018), social class (Kish-Gephart and Campbell, 2015), work experience (Terbeck et al., 2021), etc. can be referred to as sensitive periods (Kish-Gephart and Campbell, 2015; Mathias et al., 2015). For organizations, sensitive periods are likewise not only considered to be the early years of a firm’s existence, but clearly other sensitive periods can occur during the lifetime of an organization, these include discontinuities in product and factor markets (Horn et al., 2021; Nykänen, 2021), new market entry (Benner and Tripsas, 2012), periods of underperformance or crisis (Beltratti and Stulz, 2012; Schaub and Schmid, 2013), etc.

Similarly, for the SARS outbreak in 2003, this sudden public health event not only dealt a heavy blow to companies, but also created a crisis in the overall economic and business environment. We believe that the period when companies experience a SARS pandemic can be considered a “sensitive period” for companies, after which they are imprinted with the SARS imprinting, which will have a lasting impact on their subsequent survival and development. There is still a paucity of academic research on SARS imprinting, with only two papers in the available literature examining SARS imprinting. One is a country-level study of SARS imprinting, Ru et al. (2021) found that countries with a SARS imprint had a faster response time when experiencing the COVID-19 pandemic than countries without a SARS imprint, and that governments in countries without SARS were much slower to implement containment measures than those in countries that experienced SARS. Another study of SARS imprinting at the individual level, Yao et al. (2021) showed that SARS imprinting increased individuals’ fear of COVID-19 and that this effect was reduced with the use of AI and big data. Thus, according to imprinting theory, we believe that the “characteristics” of a company that are developed during the sensitive period to adapt to a particular environment have a lasting impact on subsequent decisions.

### SARS Imprinting and Firms’ Recovery During the COVID-19 Pandemic

We predict that firm without such prior experience will be far less recover after a COVID-19 pandemic outbreak than those that have experienced a similar pandemic and have similar experiences.

The literature demonstrates that identifying and mitigating the threats posed by a crisis is a key factor in achieving business continuity (Ambulkar et al., 2015). Business continuity is defined as “the ability of an organization to continue to deliver a product or service at an acceptable level after a disruptive event.” A company demonstrates business continuity if it maintains revenue streams, sustains employment, provides services to customers and maintains the confidence of shareholders, stakeholders and the public throughout a crisis (Bhamra et al., 2011). It has been argued that business continuity management contributes to firm recovery by integrating the ability to identify and mitigate threats into a firm’s day-to-day operations (Morrow et al., 2007).

As the SARS outbreak in 2003 had a high degree of similarity to the COVID-19 pandemic, both SARS and COVID-19 outbreaks were severe respiratory infections and belonged to the same family of coronaviruses, with similar prevention, control and recovery measures (Yang et al., 2020), it is possible that companies experienced SARS as an imprint of prior experience. it is possible that after gaining experience people always try to select the option that led to the best outcomes in a small sample of similar situations in the past (Erev et al., 2022). After a COVID-19 pandemic outbreak, companies are exposed to a risky environment that can lead to irreversible losses if not handled properly. Scholars have demonstrated that prior experience with similar events affects firms’ risk aversion (Bissoondoyal-Bheenick et al., 2021) and are more sensitive to the consequences of risk. Firms that have experienced SARS and recovered from the epidemic may gain knowledge from practical experience to improve their management strategies (Alonso and Kok, 2018), while developing a higher risk tolerance, becoming more cautious in their decision making choices in relation to corporate risk, becoming more conservative, and keeping their capital structure in a more reasonable state, which is conducive to improving their overall recovery when they experience a similar epidemic again. The overall recovery of the firm in the event of another similar epidemic. From the perspective of organization managers, it is possible that after gaining experience people always try to select the option that led to the best outcomes in a small sample of similar situations in the past. Managers with similar prior experience with SARS tend to quickly assess the risks that their organization will face after a COVID-19 pandemic outbreak and initiate a reasonably effective response (Bhamra et al., 2011), and when organizational decision-makers realize that their company is facing a similarly severe disaster, they can initiate anticipatory strategies. In addition, having had experience in recovering from the SARS disaster, managers may not be plagued by pessimism when faced with a COVID-19 pandemic (Bernile et al., 2017), and they will perceive a strong control over the crisis event, leading to an orderly resumption of normal company operations. From the perspective of employees, some employees in companies with the SARS imprinting have also experienced the post-SARS firms’ recovery process, and these employees tend to demonstrate a higher level of alertness, a stronger sense of apprehension, and a sense of crisis or urgency, which will make them more understandingly supportive of and cooperative with swift and rigorous firms’ recovery measures, thereby enhancing firms’ recovery (Faeni et al., 2022). All of the above arguments demonstrate that firms with the SARS imprinting exhibit greater business continuity through the COVID-19 pandemic, resulting in better retention of business performance and better recovery when faced with a new crown crisis. In summary, we conclude that,

H1: *The SARS imprinting is positively related to the firms’ recovery during the COVID-19 pandemic.*

### Moderating Role of SARS Severity

According to imprint theory, we further argue that the relationship between firms with SARS experience and recovery during the COVID-19 pandemic depends on the strength of the SARS imprint. The strength of the imprint reflects the extent to which the imprint influences individual behavior (Simsek et al., 2015). That is, if a firm has a strong SARS imprint, then its influence will increase. The imprinting literature suggests that the strength of imprinting is determined by individual characteristics during the imprint formation phase and environmental characteristics during the imprint persistence phase (Tilcsik, 2014). In our context, the severity of the impact of SARS at a firm’s location can be a good indicator of the firm’s characteristics during the imprint formation phase. Considering the intensity of a firm’s SARS imprint at the time of its formation, a firm will develop a more profound SARS imprint if the number of people diagnosed in the firm’s city during the SARS pandemic is higher, that is, the deeper the city is affected by the SARS pandemic, the more profound SARS imprint will be formed.

Firms can look for guidance on new issues in past experience, and in-depth experience can provide better solutions to these issues (Furr, 2019), and it involves the richness of a firm’s understanding of the post-crisis recovery process. From the perspective of knowledge accumulation, as companies gained deeper experience from SARS, they then made great efforts to maintain normal operations during SARS, and in the process, the emergency response models and methods established by companies were embedded in the daily life and behavior of the organization (Kang et al., 2019). In response to the COVID-19 pandemic, companies achieved repeated learning by combining past experiences as well as current new knowledge, which was more likely to help them achieve rapid recovery during the COVID-19 pandemic due to path dependency and repeated learning minimizing the time and cost of learning for companies and guiding the sequence of actions (Surdu et al., 2018).

Overall, firms located in areas with more severe SARS pandemic formed a stronger imprint than firms in areas with less severe SARS pandemic and were therefore more conducive to firm recovery during the COVID-19 pandemic. In summary, we predict that,

H2: *The more severe the SARS pandemic experienced by a firm, the more significant the effect of the SARS imprint on the firms’ recovery ability during the COVID-19 pandemic.*

### Moderating Role of Digitalization Levels

We argue that a high level of digitalization in a firm during the COVID-19 pandemic will further stimulate the SARS imprint of the firm and enhance its utility while it lasts.

Given the high level of uncertainty and ambiguity that a COVID-19 pandemic creates for organizations, it is imperative that firms are prepared with the necessary resources and accurately anticipate challenges to meet the demands of a disruptive event. While past experience has kept the capital structures of SARS-imprinted companies in a more reasonable state during a COVID-19 pandemic, the financial reserves of companies may still be depleted due to the significant and lasting negative effects of COVID-19. In contrast, digital technologies such as artificial intelligence, big data, cloud computing, blockchain and industrial internet can enhance the role of corporate SARS imprints on firms’ recovery during a COVID-19 pandemic with their unique characteristics (He et al., 2022).

On the one hand, digital technologies can increase the efficiency of firms’ use of external resources. Contingency theory suggests that organizational effectiveness is a product of the adaptation of a firm’s strategy to its surroundings, that there is no static management style, and that organizations that adapt to their environment achieve good results (Kim et al., 2014). Although experiencing SARS can leave a lasting legacy of management styles and decision-making characteristics within a organization, as the SARS pandemic was 17 years after the COVID-19 pandemic outbreak, the external resources that a organization had built up at the time have been depleted over time or are no longer adaptable to the current environment, and digital technology can improve a firm’s utilization of external resources, thereby seizing new opportunities to sustain business operations. Research has shown that digital technologies can help organizations build networks of connections with other companies, providing useful information and resources to sustain operations and build recovery in the face of disruptive crises (Chowdhury et al., 2019).

On the other hand, companies that are more digitally advanced can more easily and flexibly coordinate the internal resources that the company has integrated based on the SARS imprint to continue operations in the face of adversity (Klein and Todesco, 2021). During the COVID-19 pandemic, many companies were forced to shut down their operations due to quarantine related policies, but companies that invested in digital technology could continue operations through innovative ways to enable interaction with customer interaction to continue operations. In addition, digital information technology enabled companies to offer alternative plans for work when many of their employees were isolated at home (Lee et al., 2022). In summary, we propose the following hypothesis,

H3: *The higher the level of digitalization of a firm, the more significant the effect of the SARS imprint on the firm’s recovery during the COVID-19 pandemic.*

## Materials and Methods

### Sample and Data Sources

Our sample data comes from the CSMAR database. We focus on A-share companies listed on the Shanghai and Shenzhen stock exchanges, excluding special treatment (ST) companies. Our sample period is 2020, with a statistical cycle of one quarter to create the panel data. Since the COVID-19 pandemic outbreak in China occurs in December 2019 and enters the pandemic phase in 2020. The firm recovery examined in this study focuses on the ability of firms to recover from a crisis when they experience an outbreak, and the year 2020 is on the rise of the pandemic that better highlights the ability of firms to deal with a crisis. Currently, although the COVID-19 pandemic is still ongoing, firms have formed a normal management system after a year of adjustment and consolidation, studying firm recovery at this time may not be representative, thus biasing the impact of the SARS imprint on firm recovery ability. Therefore, we believe that 2020 is more appropriate to test the crisis recovery ability of firms and using 2020 as a sample window can lead to clearer conclusions on our research questions. To make the analysis below more accurate and credible, we treat the sample as follows: (1) remove the sample of ST and *ST companies; (2) remove the sample of companies with incomplete data for the current period; we end up with 16,818 sample observations, involving a total of 4,753 listed companies. To eliminate the effect of extreme values, we apply tailoring to the main continuous variables at the upper and lower 1% levels.

### Measures

#### Dependent Variable

The firms’ recovery discussed in this study focuses on the performance recovery of enterprises. Previous literature has discussed the turnover and recovery cycle of enterprises after crisis (Jin et al., 2018). These studies focus on the time required for enterprises to resume normal operation, and we focus on the recovery ability of enterprises. In order to further determine the measurement standard of enterprise performance recovery, we refer to the research of peers. We find that most studies use profitability to express performance. For example, some scholars regard ROA as a measure of enterprise performance. Minichilli et al. (2016) investigated the recovery of business performance of family enterprises in the event of financial and economic crisis. They pointed out that ROA, as a measure of short-term accounting performance, seems to be particularly suitable to represent the short-term financial performance and long-term objectives of enterprises. Barbero et al. (2020) defined a company in decline as one that has two consecutive years of declining ROA when studying the relationship between renewal aggressiveness and turn-around performance. Other scholars use other measurement indicators. For example, Kettunen et al. (2021) believe that if the firm becomes profitable next fiscal year, it is considered to have achieved turnover. Early scholars used return on investment (Chowdhury et al., 1996; O’Kane and Cunningham, 2014), ratio of income as percentage of sales (ROS; Barker and Mone, 1994), or market share (Thietart, 1988). Considering the availability of data, we choose to return on assets (ROA) as the proxy variable of enterprise recovery. The ROA is calculated as the ratio of net profit to the average balance of total assets, which is usually used to evaluate the impact of ownership and governance characteristics on corporate performance and family business research (Salvato et al., 2020), and to measure the profitability of private enterprises (Fujii et al., 2013). In addition, in order to understand whether the change of ROA is driven by operating profit, we also use the logarithm of operating revenue growth rate as the dependent variable for robustness test.

#### Independent Variables

Our dependent variable is whether the firm is imprinted with SARS. A firm is considered to be imprinted with SARS if it was established prior to the SARS outbreak and experienced the SARS pandemic. We coded firms established before 2003 as 1 and firms established after 2003 as 0.

#### Moderating Variables

The first moderating variable in this paper is the severity of SARS (H2), which moderates the intensity of a firm’s SARS imprint when it is formed. We argue that if a firm is located in a city where more people were diagnosed with SARS during the SARS outbreak, the more deeply that city was affected by the SARS pandemic. Therefore, we used the number of confirmed SARS cases to measure the severity of SARS at the business location. In our sample, the city most affected by SARS was Beijing, which accounted for 47.3% of all confirmed cases nationwide.

The second moderating variable in this paper is a firm’s level of digitalization (H3), which is used to moderate the intensity of a firm’s SARS imprint during the duration phase. As the importance that firms place on a particular strategic orientation can often be reflected by the frequency with which the keywords involved in that strategy appear in their annual reports. In this regard, we used the sum of the frequency of keyword terms referring to artificial intelligence technology, blockchain technology, cloud computing technology, big data technology, and digital technology applications collected in the CSMAR database in companies’ annual reports to measure the level of digitalization of companies.

#### Control Variables

We controlled for factors commonly used in the relevant literature to influence firm performance. First, controlling for employee size, research has shown that employees are an important foundation of a business and that employee recovery and labor efficiency have a significant impact on firm recovery (Liang and Cao, 2021). During the COVID-19 pandemic, the entire economy and society was threatened by the spread of the disease and infection, and the attitudes and behaviors of the firm’s employees were affected by this particular event, which in turn affected the firm’s production and operations. We also control for liquidity ratios and gearing ratios. As financial elements are one of the material bases for the survival and development of enterprises, working capital, of which cash flow, accounts payable and short-term borrowing are important components, reflects whether a company has sufficient funds to cover various operating expenses that may be incurred on a daily basis and to ensure its continued operation (Orlando et al., 2022), we focused on the liquidity ratios and gearing ratios. In addition, we further controlled for a number of corporate governance characteristics and financial conditions, including firm size, registered capital, Tobin’s Q, and the number of board meetings held. We also included industry and region dummy variables to control for differences due to industry and region.

### Econometric Approach

In this section, we examine the impact of a firm’s SARS imprint on a firm’s corporate performance recovery following a COVID-19 pandemic. As discussed above, firms are imprinted with the SARS if they were established prior to the SARS outbreak, and this imprint positively affects a firm’s recover ability after the COVID-19 pandemic (H1). Secondly, if a firm experienced a more severe SARS pandemic in that year, it would deepen the SARS imprint on the firm, which will be more conducive to enhancing the firm’s recovery (H2). Finally, if the firm was undergoing or completing a digital transformation during the COVID-19 pandemic and improved its own digitalization, it would be more conducive to improving its own recovery during the epidemic (H3). In summary, we developed the following OLS model for baseline regressions.

$${\text{Recovery}}_{\text{roa}}=\alpha_{0}+\alpha_{1}\,\mathrm{Before}\,{\mathrm{SARS}}_{\text{it}}+\alpha_{2}\,\mathrm{Before}\,{\mathrm{SARS}}_{\text{it}}\times \mathrm{SARS}\,{\mathrm{confirmed}}_{\text{it}}+\alpha_{3}\,\mathrm{Before}\,{\mathrm{SARS}}_{\text{it}}\times {\text{Digital}}_{\text{it}}+\alpha_{4}\,\mathrm{SARS}\,{\mathrm{confirmed}}_{\text{it}}+\alpha_{5}\,{\text{Digital}}_{\text{it}}+\sum {\text{Control}}_{\text{it}}+\delta_{t}+\mu_{\text{it}}$$

where *i* represents firm, *t* is the time, μ_it_ is the standard error term, and δ_*t*_ is the time fixed effect. Table 1 defines all the variables.

**TABLE 1:** Variable definition.

Variables	Definition
Recovery_roa	We use ROA to measure the recovery of firms. ROA is calculated as the ratio of net income to total asset balance.
Before SARS	A firm is considered to be imprinted with SARS if it was established prior to the SARS outbreak and experienced the SARS pandemic. We coded firms established before 2003 as 1 and firms established after 2003 as 0.
Regcap	Registered capital.
Board meeting	Number of board meetings.
Firm size	Total assets of listed firms at the beginning of the year.
Q	Ratio of market capitalization to total assets.
Employee	Total number of employees in listed firms.
Current	Ratio of total current assets to total assets.
Debt	Ratio of total liabilities to total assets.
Digital	Firm’s level of digitalization.
SARS severity	We used the number of confirmed SARS cases to measure the severity of SARS at the firms location.

## Results

### Descriptive Statistics

To ensure that multicollinearity did not affect the results, we calculated variance inflation factors (VIFs), which are indicators of covariance between predictors. The results showed that the VIFs for all variables were below 4.6 (mean = 1.76), well below the generally accepted threshold of 10.0 (Neter et al., 1996). We also tested the correlation coefficients between the variables, with the highest value of 0.65 being below 0.70, which is the minimum that is considered possible for multicollinearity (Kennedy, 2003). Table 2 shows the correlation coefficients and descriptive statistics for all variables, except for the industry and region dummy variables.

**TABLE 2:** Descriptive statistics and correlations.

Variables	Obs	Mean	S.D.	(1)	(2)	(3)	(4)	(5)	(6)	(7)	(8)	(9)	(10)	(11)
(1) Recovery_roa	10,560	0.027	0.074	1.000										
(2) Before SARS	10,560	0.732	0.443	0.034	1.000									
(3) Regcap	10,560	20.040	1.079	−0.134	0.194	1.000								
(4) Board meeting	10,560	2.319	0.355	−0.085	0.016	0.226	1.000							
(5) Firm size	10,560	22.273	1.383	−0.020	−0.008	0.289	0.056	1.000						
(6) Q	10,560	2.101	1.764	0.137	−0.064	−0.172	−0.055	−0.044	1.000					
(7) Employee	10,560	7.742	1.283	−0.015	−0.007	0.432	0.118	0.645	−0.071	1.000				
(8) Current	10,560	0.600	0.195	0.120	−0.135	−0.337	−0.107	−0.056	0.109	−0.083	1.000			
(9) Debt	10,560	0.412	0.226	−0.213	0.113	0.343	0.262	0.135	−0.205	0.186	−0.122	1.000		
(10) Digital	10,560	2.219	1.157	−0.011	−0.065	−0.004	0.051	0.092	0.068	0.076	0.200	−0.038	1.000	
(11) SARS severity	10,560	3.705	2.629	−0.024	−0.057	0.040	0.127	0.079	0.020	0.129	0.048	0.006	0.150	1.000

### Hypotheses Testing

Table 3 presents the OLS estimation results of the model of the relationship between corporate SARS imprinting and firms’ recovery during the COVID-19 pandemic.

**TABLE 3:** OLS regression results of the relationship between SARS imprinting and firms’ recovery during the COVID-19 pandemic.

	Model 1	Model 2	Model 3	Model 4
Variables	Recovery_roa	Recovery_roa	Recovery_roa	Recovery_roa
Before SARS		0.0172***	0.0145***	0.0170***
		(0.0016)	(0.0016)	(0.0016)
Before SARS ×			0.0017***	
SARS severity			(0.0007)	
Before SARS ×				0.0463**
Digital				(0.0227)
Regcap	−0.0030	−0.0040	−0.0010	−0.0040
	(0.0025)	(0.0025)	(0.0024)	(0.0025)
Board meeting	−0.0068***	−0.0053**	0.0031	−0.0053**
	(0.0026)	(0.0026)	(0.0028)	(0.0026)
Firm size	−0.0014	−0.0014	0.0010	−0.0014
	(0.0016)	(0.0016)	(0.0016)	(0.0016)
Q	0.0039***	0.0041***	0.0060***	0.0041***
	(0.0009)	(0.0009)	(0.0008)	(0.0008)
Employee	0.0075***	0.0077***	0.0035**	0.0077***
	(0.0014)	(0.0014)	(0.0014)	(0.0014)
Current	0.0370***	0.0405***	0.0378***	0.0403***
	(0.0049)	(0.0048)	(0.0049)	(0.0048)
Debt	−0.0511***	−0.0532***	−0.0503***	−0.0532***
	(0.0080)	(0.0080)	(0.0083)	(0.0080)
Digital	−0.0009	−0.0009	0.0006	−0.0016*
	(0.0007)	(0.0007)	(0.0008)	(0.0008)
SARS severity	−0.0093	−0.0118	0.0183*	−0.0117
	(0.0187)	(0.0188)	(0.0099)	(0.0188)
Constant	0.0715***	0.0651**	−0.0357	0.0673***
	(0.0255)	(0.0253)	(0.0260)	(0.0253)
City dummies	Yes	Yes	Yes	Yes
Industry dummies	Yes	Yes	Yes	Yes
Observations	10,560	10,560	10,554	10,560
Pseudo *R*^2^	0.1187	0.1274	0.1208	0.1276

*Standard errors are in parentheses.*

***p < 0.01, **p < 0.05, and *p < 0.1.

Models 1–4 examine the effect of corporate SARS imprinting on firms’ recovery (*Recovery_roa*) during the COVID-19 pandemic. Model 1 reports the results of the baseline model that includes only control and moderating variables. In Model 2, we include the independent variable, SARS imprint, and the results show a significant positive effect of a firm’s SARS imprint on firm recovery during the COVID-19 pandemic (α_1_ = 0.0172, *p* < 0.01), providing support for Hypothesis 1. Hypothesis 2 posits that the deeper the location of a firm is affected by the SARS pandemic, the deeper the firm’s SARS imprint, i.e., the severity of SARS has a moderating effect on the relationship between a firm’s SARS imprint and firm recovery during the COVID-19 pandemic. As shown in Model 3, the coefficient on the interaction term between SARS severity and firm SARS imprint was positive and statistically significant (α_2_ = 0.0017, *p* < 0.01), indicating that the relationship between firm SARS imprint and firm recovery during the COVID-19 pandemic increased as SARS severity deepened, with results strongly supporting Hypothesis 2. Hypothesis 3 predicts that the level of corporate digitalization moderates the relationship between corporate SARS imprint and firms’ recovery during the COVID-19 pandemic. Model 4 showed that the coefficient of the interaction term between the level of corporate digitization and corporate SARS imprint was positive and statistically significant (α_3_ = 0.0463, *p* < 0.5), indicating that the stronger the level of corporate digitisation, the more the SARS imprint contributed to firms’ recovery during the COVID-19 pandemic.

## Robustness Checks

### Other Indicators of Firms’ Recovery

As described in the previous sections, we use the growth rate of operating income (*Recovery_income*) as a proxy for the recovery of firms. Table 4 shows the results of the regression between the SARS imprint and the growth rate of operating income. The results show that the SARS imprint of a firm still has a significant positive effect on the growth rate of operating income, and that the severity of SARS and the level of digitization of the firm still positively moderate the relationship between the SARS imprint and the firm’s recovery. This proves that our baseline regression results are robust. Slightly different from the baseline regression results, the impact of the SARS imprint of the firm on the growth rate of operating income is stronger than the impact on the ROA.

**TABLE 4:** Results from the other indicators of firms’ recovery.

	Model 1	Model 2	Model 3	Model 4
Variables	Recovery_income	Recovery_income	Recovery_income	Recovery_income
Before SARS		0.116***	0.1105***	0.1149***
		(0.0102)	(0.0103)	(0.0103)
Before SARS ×			0.0135***	
SARS severity			(0.0037)	
Before SARS ×				0.3662**
Digital				(0.1826)
Regcap	−0.0152**	−0.0211***	−0.0217***	−0.0210***
	(0.0072)	(0.0071)	(0.0071)	(0.0071)
Board meeting	0.0239*	0.0334**	0.0319**	0.0333**
	(0.0134)	(0.0134)	(0.0135)	(0.0134)
Firm size	−0.0091	−0.0094	−0.0095	−0.0097
	(0.0098)	(0.0098)	(0.0098)	(0.0098)
Q	0.0262***	0.0278***	0.0277***	0.0279***
	(0.0026)	(0.0026)	(0.0026)	(0.0026)
Employee	0.0334***	0.0350***	0.0357***	0.0353***
	(0.0087)	(0.0086)	(0.0086)	(0.0086)
Current	0.0768**	0.0953***	0.0954***	0.0939***
	(0.0337)	(0.0335)	(0.0335)	(0.0335)
Debt	0.0922***	0.0807***	0.0826***	0.0805***
	(0.0274)	(0.0270)	(0.0273)	(0.0269)
Digital	0.0139***	0.0140***	0.0138***	0.0084
	(0.0050)	(0.0049)	(0.0049)	(0.0061)
SARS severity	0.0044	−0.0073	−0.0199	−0.0067
	(0.0653)	(0.0671)	(0.0664)	(0.0670)
Constant	−0.1276	−0.1776	−0.1163	−0.1607
	(0.1949)	(0.1925)	(0.1948)	(0.1919)
City dummies	Yes	Yes	Yes	Yes
Industry dummies	Yes	Yes	Yes	Yes
Observations	10,151	10,151	10,151	10,151
Pseudo *R*^2^	0.1940	0.2043	0.2054	0.2046

*Standard errors are in parentheses.*

***p < 0.01, **p < 0.05, and *p < 0.1.

### Endogeneity Tests

Considering the many similarities between the COVID-19 pandemic and the SARS pandemic that broke out in 2003, it is inevitable that firms with the SARS imprint will use the recovery measures and methods used during SARS in that year when responding to the COVID-19 pandemic, which may cause firms to revert to or deepen their management philosophy and approach from that year, thus creating an error term in our model μ_it_ correlates with the firm’s SARS imprint, leading to a reverse causality relationship and creating a serious endogeneity problem.

To deal with the reverse causality between the SARS imprinting and firms’ recovery during the COVID-19 pandemic, we used the Heckman two-stage model (Heckman, 1979). The first stage uses a Probit regression model in which the dependent variable Before SARS is a dummy variable that equals 1 if the firm was established before the SARS pandemic outbreak and 0 otherwise. Since the area most severely affected by the SARS pandemic at the time of the outbreak in 2003 was Beijing, whose cumulative confirmed cases accounted for nearly half of the confirmed cases nationwide. Therefore, we argue that enterprises established before 2003 are located closer to Beijing, the more profound the SARS imprint of the enterprise, and that this geographical distance does not affect the ability of the enterprise to recover during the COVID-19 pandemic. We therefore chose the geographical distance between the firm’s city of location and Beijing as an additional binding variable to include in the first stage regression and calculated the inverse Mills ratio (*IMR*). This constraint variable is derived from historical data from 2003 and does not directly affect the current firm’s recovery, satisfying the exogeneity requirement of the constraint variable. Panel A of Table 4 shows the regression results for the first stage.

Next, we use *Before SARS* as the dependent variable and substitute the *IMR* obtained in the first stage regression into the second stage regression to control for endogeneity issues in the main model during the regression. Panel B of Table 5 reports the results of the second-stage regression. In the second stage, the modified *Before SARS*, *Before SARS SARS* confirmed and Before *SARS Digital* impact mechanisms remain the same as in the previous main effects regressions, indicating that after excluding endogeneity issues, the impact of corporate SARS imprinting on corporate during the COVID-19 pandemic The enhancement effect of recovery remains. It can be seen that the findings of this paper are still significant after excluding the potential endogenous issues.

**TABLE 5:** Results from the Heckman two-stage model.

	A: Heckman I	B: Heckman II			
	Model 1	Model 2	Model 3	Model 4	Model 5
			
Variables	Before SARS	Recovery_roa	Recovery_roa	Recovery_roa	Recovery_roa
Before SARS			0.0176***	0.0168***	0.0174***
			(0.0016)	(0.0017)	(0.0017)
Before SARS ×				0.0019***	
SARS severity				(0.0006)	
Before SARS ×					0.0514**
Digital					(0.0228)
Distance	0.6262				
	(0.3815)				
Regcap	0.1636***	0.0003	0.0005	0.0004	0.0005
	(0.0233)	(0.0031)	(0.0031)	(0.0031)	(0.0031)
Board meeting	−0.3204***	−0.0134***	−0.0137***	−0.0141***	−0.0138***
	(0.0479)	(0.0040)	(0.0040)	(0.0040)	(0.0040)
Firm size	0.0160	−0.0014	−0.0014	−0.0014	−0.0014
	(0.0265)	(0.0017)	(0.0017)	(0.0017)	(0.0017)
Q	−0.0330***	0.0029***	0.0029***	0.0028***	0.0029***
	(0.0086)	(0.0008)	(0.0008)	(0.0008)	(0.0008)
Employee	−0.0491**	0.0069***	0.0068***	0.0069***	0.0069***
	(0.0207)	(0.0015)	(0.0015)	(0.0015)	(0.0015)
Current	−0.6564***	0.0237***	0.0230***	0.0226***	0.0227***
	(0.0980)	(0.0079)	(0.0079)	(0.0079)	(0.0079)
Debt	0.5080***	−0.0433***	−0.0429***	−0.0425***	−0.0429***
	(0.0918)	(0.0097)	(0.0097)	(0.0097)	(0.0097)
Digital	−0.0035	−0.0012*	−0.0012*	−0.0013*	−0.0021**
	(0.0156)	(0.0007)	(0.0007)	(0.0007)	(0.0009)
SARS severity	0.8087*	0.0116	0.0114	0.0098	0.0116
	(0.4536)	(0.0291)	(0.0297)	(0.0296)	(0.0297)
IMR		0.0407***	0.0546***	0.0556***	0.0547***
		(0.0154)	(0.0157)	(0.0157)	(0.0157)
Constant	−6.731***	0.0733	0.0533	0.0610	0.0555
	(2.478)	(0.0660)	(0.0674)	(0.0669)	(0.0675)
City dummies	Yes	Yes	Yes	Yes	Yes
Industry dummies	Yes	Yes	Yes	Yes	Yes
Observations	9,298	9,270	9,270	9,270	9,270
Pseudo *R*^2^		0.0976	0.1070	0.1079	0.1072

*Standard errors are in parentheses.*

***p < 0.01, **p < 0.05, and *p < 0.1.

### Heterogeneity Test

SARS pandemic and COVID-19 pandemic are different in scale and scope of influence, and the impact of SARS on different industries and different types of firms is also different. In order to identify the impact of differentiation caused by differences in industry or firm characteristics, we will examine the impact of SARS imprint on the recovery of small and medium-sized firms, large firms, state owned firms, private owned firms and foreign owned firms, as well as the impact of SARS imprinting on the recovery of transportation, storage and postal services, information transmission, software and information technology services, manufacturing, construction, real estate, wholesale and retail, culture, sports and entertainment, and finance industries.

#### Firm Heterogeneity

Table 6 shows the impact of SARS imprint on the recovery of different types of firms. Models 1 and 2 examined the differences in the recovery of firms of different sizes in the COVID-19 pandemic. The results show that the SARS imprint is conducive to the recovery of firms in COVID-19 pandemic. However, the impact of SARS imprint on large firms is lower than that of small and medium-sized firms. Generally, the larger the firm, the greater the structural inertia it may cause in the recovery process of COVID-19 pandemic. As large firms involve too many stakeholders, once management practices are formed, it is often difficult to change operation decisions and behaviors. Therefore, for firms with large structural inertia, there is less room for their own SARS experience. Model 3, Model 4, and Model 5 investigate the difference of recovery ability of firms with different equity properties in COVID-19 pandemic. As shown in Table 6, SARS imprint has a significant positive impact on the recovery of three types of firms. The difference is that SARS imprint has the greatest impact on the recovery ability of private firms, followed by foreign-funded firms and finally state-owned firms. This result is in line with our expectations.

**TABLE 6:** The impact of SARS imprint on the recovery of different types of enterprises.

Variables	Model 1	Model 2	Model 3	Model 4	Model 5
	Small and medium-sized enterprises	Large enterprises	State-owned enterprise	Private-owned enterprise	Foreign-owned enterprise
Before SARS	0.0197***	0.0075***	0.0057**	0.0217***	0.0162***
	(0.0018)	(0.0018)	(0.0023)	(0.0023)	(0.0059)
Regcap	−0.0037	−0.0003	−0.0041***	−0.0038	0.0002
	(0.0025)	(0.0010)	(0.0011)	(0.0039)	(0.0046)
Board meeting	−0.0056**	−0.0034*	−0.0022	−0.0083*	0.0120
	(0.0027)	(0.0019)	(0.0022)	(0.0044)	(0.0091)
Firm size	−0.0012	0.0005	0.0076***	−0.0039*	−0.0134**
	(0.0017)	(0.0014)	(0.0014)	(0.0023)	(0.0056)
Q	0.0044***	0.0065***	0.0089***	0.0035***	0.0077***
	(0.0009)	(0.0006)	(0.0011)	(0.0012)	(0.0014)
Employee	0.0075***	0.0072***	−0.0001	0.0103***	0.0214***
	(0.0014)	(0.0013)	(0.0012)	(0.0025)	(0.0044)
Current	0.0389***	0.0370***	0.0155***	0.0529***	0.0139
	(0.0051)	(0.0041)	(0.0053)	(0.0080)	(0.0154)
Debt	−0.0533***	−0.0600***	−0.0460***	−0.0534***	−0.0669***
	(0.0082)	(0.0060)	(0.0063)	(0.0095)	(0.0199)
Digital	−0.0008	0.0010	0.0007	−0.0025**	−0.0048
	(0.0007)	(0.0008)	(0.0009)	(0.0012)	(0.0031)
SARS confirmed	−0.0106	−0.0354***	0.0104	−0.0066	0.0072
	(0.0189)	(0.0109)	(0.0091)	(0.0280)	(0.0199)
Constant	0.0530**	0.0166	−0.0606**	0.1026**	0.0982
	(0.0264)	(0.0268)	(0.0275)	(0.0435)	(0.1089)
City dummies	Yes	Yes	Yes	Yes	Yes
Industry dummies	Yes	Yes	Yes	Yes	Yes
Observations	10,162	5,384	3,021	6,328	712
R-squared	0.1283	0.2969	0.3616	0.1197	0.5538

*Standard errors are in parentheses.*

***p < 0.01, **p < 0.05, and*p < 0.1.

#### Industry Heterogeneity

Table 7 shows the impact of SARS imprint on the recovery of firms in different industries. The results showed that except for wholesale and retail, SARS imprint had a significant positive impact on the recovery of other industries. Among them, the most significant impact is real estate and culture, sports and entertainment, followed by construction. It can be seen that real estate and construction firms are better at applying the early experience to the current operation and management. However, we found that SARS imprint had little effect on manufacturing. The reason may be that manufacturing is an industry related to the national economy and the people’s livelihood. When the SARS pandemic broke out, with the support of national policies, manufacturing firms were less impacted by the pandemic and did not form a profound imprint of SARS. In addition, the SARS imprint also did not have a great impact on finance. The financial industry is greatly affected by the macroeconomic environment. Since SARS broke out in 2003, it has been 17 years since 2020. During these 17 years, great changes have taken place in China’s macro environment, market-oriented level and regulatory system. The management experience formed in that year may no longer adapt to the current environment.

**TABLE 7:** The impact of SARS imprint on the recovery of enterprises in different industries.

Variables	Panel A			
	Model 1	Model 2	Model 3	Model 4
	Transportation, storage and postal services	Information transmission, software and information technology services	Manufacturing	Construction
Before SARS	0.0174***	0.0315***	0.0190***	0.0406***
	(0.0046)	(0.0048)	(0.0020)	(0.0069)
Regcap	0.0000	−0.0082***	−0.0093***	0.0025
	(0.0042)	(0.0025)	(0.0018)	(0.0044)
Board meeting	0.0033	−0.0064	−0.0006	0.0130
	(0.0086)	(0.0062)	(0.0021)	(0.0096)
Firm size	−0.0034	−0.0095***	−0.0015	−0.0025
	(0.0045)	(0.0034)	(0.0021)	(0.0058)
Q	−0.0057	0.0016	0.0041***	0.0005
	(0.0055)	(0.0010)	(0.0007)	(0.0098)
Employee	−0.0011	0.0103***	0.0128***	0.0015
	(0.0026)	(0.0031)	(0.0014)	(0.0036)
Current	−0.0137	0.0195*	0.0424***	0.0251
	(0.0229)	(0.0111)	(0.0052)	(0.0186)
Debt	−0.0057	−0.0617**	−0.0673***	−0.0053
	(0.0270)	(0.0267)	(0.0070)	(0.0248)
Digital	−0.0015	−0.0030	−0.0029***	0.0015
	(0.0026)	(0.0021)	(0.0008)	(0.0025)
SARS confirmed	0.1958	−0.0582	−0.0085	0.0040
	(0.1473)	(0.0807)	(0.0282)	(0.0552)
Constant	−0.3246	0.4422**	0.1186***	−0.0744
	(0.3336)	(0.1938)	(0.0227)	(0.1183)
city dummies	Yes	Yes	Yes	Yes
Observations	301	1,140	6,812	240
R-squared	0.2450	0.2061	0.1818	0.2955

**Variables**	**Panel B**			
	**Model 5**	**Model 6**	**Model 7**	**Model 8**
	**Real estate**	**Wholesale and retail**	**Culture, sports and entertainment**	**Finance**

Before SARS	0.0965***	−0.0020	0.0976***	0.0133***
	(0.0323)	(0.0046)	(0.0181)	(0.0021)
Regcap	−0.0004	0.0172**	−0.0082	−0.0006
	(0.0035)	(0.0082)	(0.0118)	(0.0023)
Board meeting	−0.0095	0.0108**	−0.0279	0.0025
	(0.0087)	(0.0044)	(0.0187)	(0.0044)
Firm size	−0.0009	0.0025	0.0036	−0.0005
	(0.0030)	(0.0042)	(0.0094)	(0.0014)
Q	−0.0014	−0.0127**	0.0036	0.0023
	(0.0011)	(0.0053)	(0.0060)	(0.0025)
Employee	0.0001	0.0044*	−0.0068	0.0000
	(0.0025)	(0.0024)	(0.0066)	(0.0015)
Current	0.0051	0.0081***	−0.0015	−0.0012
	(0.0144)	(0.0025)	(0.0258)	(0.0045)
Debt	−0.0075	0.0560***	0.0406	0.0060
	(0.0215)	(0.0083)	(0.0413)	(0.0086)
Digital	0.0006	−0.0605***	0.0238*	0.0036**
	(0.0019)	(0.0104)	(0.0132)	(0.0017)
SARS confirmed	−0.0093	0.0026	0.0075	−0.0008
	(0.0097)	(0.0016)	(0.0136)	(0.0023)
Constant	−0.0119	−0.0134	0.0434	0.0017
	(0.0624)	(0.0508)	(0.1877)	(0.0467)
city dummies	Yes	Yes	Yes	Yes
Observations	248	496	192	446
R-squared	0.3869	0.3993	0.5170	0.1537

*Standard errors are in parentheses.*

***p < 0.01, **p < 0.05, and *p < 0.1.

## Discussion

Organizations are often subject to external disruptions such as natural disasters, political unrest, and pandemics. The recent COVID-19 pandemic is one of them. Such disruptions can pose a significant threat to an organization because they are often unpredictable and beyond the organization’s control. Firms cannot withstand adversity for long, so it is especially important to build recovery quickly. We look for ways to help organizations recover from similar events they have experienced in the past, and we focus on the SARS imprint of the organization, i.e., whether the organization experienced a SARS pandemic similar to COVID-19 in 2003. In conjunction with the above, we argue that a firm’s SARS experience can have an imprinting effect on a firm’s recovery during the COVID-19 pandemic.

First, a firm’s SARS experience can significantly improve a firm’s recovery during a COVID-19 pandemic. Firms that experienced SARS and recovered from the epidemic may gain knowledge from practical experience to improve their management strategies (Bissoondoyal-Bheenick et al., 2021), as well as develop higher risk tolerance, become more cautious and conservative in their decision choices related to corporate risk, and keep their capital structure in a more reasonable state, which conducive to improving the overall recovery of firms when they experience a similar epidemic again. This is reflected in our first hypothesis, where we expect the impact of firms’ SARS experience on firms’ recovery during the COVID-19 pandemic to be positive, and our empirical results support this hypothesis.

Second, we find that firms will develop a more profound SARS imprint if they experience a more severe SARS pandemic. That is, the more severe the SARS pandemic experienced by the firm, the more significant the effect of SARS experience on the firm’s recovery ability during COVID-19 pandemic. Firms can look for guidance about new problems in past experiences, and in-depth experiences can provide better solutions to these problems (Furr, 2019), and it involves the richness of firms’ understanding of the post-crisis recovery process. In this process, the emergency handling models and methods established by the firm are embedded in the daily life and behavior of the organization (Kang et al., 2019), thus helping the firm to achieve faster recovery. This hypothesis was confirmed in our empirical study.

Finally, during the COVID-19 pandemic period, the effect of SARS imprinting on firms’ recovery was more pronounced if companies were more digitized. In addition to past experiences with similar events, we also considered the impact of the current level of digitalization of the firm, a very important technological capability, on the recovery of the firm. Digital technology can help firms coordinate internal and external resources more flexibly during the COVID-19 pandemic. According to the theory of power change, there is no static management style, and organizations that adapt to their environment achieve good results (Kim et al., 2014). Digital technology can complement the SARS imprint’s missing ways and methods of adapting to the current environment, creating new external networks and seizing new opportunities to sustain business operations. Our results also confirm the positive moderating effect of the level of digitalization of the firm.

### Theoretical Contributions

Our study has several important theoretical implications. First, we contribute to the recovery literature by exploring the relationship between SARS imprinting and firms’ recovery, particularly during the COVID-19 pandemic. More studies have explored the impact of pandemic COVID-19 on individual mental health and recovery from an individual perspective (Ahmed et al., 2020), however, there are still fewer studies on firms’ recovery after the COVID-19 outbreak, with only studies from executive characteristics (Chesbrough, 2020), corporate strategy (Kong et al., 2021), technological capabilities (Doerr et al., 2021), but this literature ignores the impact of firm imprinting. Our study empirically analyzes the impact of corporate SARS imprinting on firms’ recovery during the COVID-19 pandemic, filling part of the research gap.

Second, there are still relatively few academic studies on SARS imprinting, and only two papers in the existing literature have conducted studies on SARS imprinting, one by Ru et al. (2021) on SARS imprinting at the national level and the other by Yao et al. (2021) on SARS imprinting at the individual level. To the best of our knowledge, no scholars have introduced SARS imprinting into the study of firms before our study. In our framework, we argue that a firm is imprinted with SARS at its inception if it experienced the SARS pandemic in that year, providing new insights into the application of imprinting theory in the COVID-19 pandemic period and bridging the limitations of imprinting theory in the current crisis. That is, companies can find ways to recover from a COVID-19 pandemic by looking at their past experiences with similar events.

### Practical Implications

In addition to the above theoretical contributions, our findings have practical implications for firm recovery during the COVID-19 pandemic. First, based on the crisis brought about by COVID-19 pandemic, firms may identify the importance of similar experiences in the past and the shortcomings of their organizations in terms of prevention and control systems and emergency management systems for emergencies. Companies that have not experienced the SARS pandemic can refer to the practices of companies with the SARS imprint in maintaining business operations during the COVID-19 pandemic to improve their understanding of the environment in which they operate and their alertness to crises, and to enhance their intrinsic understanding of their own cost structure in order to maintain asset balance and improve their ability to withstand crises when a major epidemic hit.

Second, accelerating the digital transformation and digital innovation of enterprises and improving their digitalization can help them achieve rapid recovery from a COVID-19 pandemic. In the face of a devastating crisis event like COVID-19, for companies that have already started digital transformation or are already digital, they can use digital technology as a fundamental resource to develop multiple communication channels with customers, suppliers, partners and stakeholders to enhance information exchange and knowledge sharing. For example, play the role of social media communication medium, big data analysis of customer needs, precision marketing and other technologies. For companies that are still on the edge of digitalization, managers can consider building the ability to respond to the crisis through corporate digitalization options. For example, in the short-term strategy can achieve a stepwise evolution in the short term by purchasing digital services or integrating themselves into the digital business ecosystem.

## Limitations and Future Research Directions

Our study has several limitations that may form the basis for future research. First, our study was conducted within a small-time frame (4 quarters), a time horizon that may not be sufficient to account for the long-term impact of SARS imprinting on the improvement of firms’ recovery, particularly regarding the path dependence of firms’ recovery. Future research could examine firms’ recovery over a longer time horizon, spanning different crises and examining individual and organizational recovery as the environment allows. This contributes to a more comprehensive understanding of individual and organizational-level change and development, and the related building of recovery. Second, our study was conducted in a single national setting (i.e., China), which means that there are qualities that may not be applicable to other settings. Future research could be conducted in other settings to be able to draw clear conclusions about the relevance of the organization-imprint relationship in the study of firms’ recovery. In addition, through cross-country studies and comparisons, future work could understand which micro foundations, relationships, and influences are relevant to the study of firms’ recovery. Finally, our selection of a sample involving firms that survived the SARS pandemic implies a potential survivorship bias, which may lack insight into failed firms. Future research could utilize a different sample, or study surviving and failing firms differently, to understand whether there are fundamental differences in the underlying picture of recovery and processes between the two samples.

## Data Availability Statement

The original contributions presented in the study are included in the article/supplementary material, further inquiries can be directed to the corresponding author.

## Author Contributions

JW drafted the manuscript. HY provided the writing instruction. JW and ML collected, analyzed, and interpreted the data. QB instructed the project. All authors contributed to the article and approved the submitted version.

## Conflict of Interest

The authors declare that the research was conducted in the absence of any commercial or financial relationships that could be construed as a potential conflict of interest.

## Publisher’s Note

All claims expressed in this article are solely those of the authors and do not necessarily represent those of their affiliated organizations, or those of the publisher, the editors and the reviewers. Any product that may be evaluated in this article, or claim that may be made by its manufacturer, is not guaranteed or endorsed by the publisher.
